# F2 peptide fraction of *Androctonus crassicauda* scorpion venom: Inducing M2 to M1 macrophage polarization and inhibiting colon carcinoma cell proliferation and migration

**DOI:** 10.22038/ajp.2025.25721

**Published:** 2025

**Authors:** Nooshin Ghadiri, Mohammad Rashno, Ali Khodadadi, Ali Asadirad, Mohammad Nemati, Ata A. Ghadiri

**Affiliations:** 1 *Cellular and Molecular Research Center, Ahvaz Jundishapur University of Medical Sciences, Ahvaz, Iran*; 2 *Department of Immunology, Faculty of Medicine, Ahvaz Jundishapur University of Medical Sciences, Ahvaz, Iran*; 3 *ancer, Petroleum and Environmental Pollutants Research Center, Ahvaz Jundishapur University of Medical Sciences, Ahvaz, Iran*; 4 *Department of Venomous Animals and Anti-Venom Production, Razi Vaccine and Serum Research Institute, Agricultural Research, Education and Extension Organization (AREEO), Ahvaz, Iran*; 5 *Air Pollution and Respiratory Diseases Research Center, Ahvaz Jundishapur University of Medical Sciences, Ahvaz, Iran*

**Keywords:** Colorectal cancer, Scorpion venom, Androctonus crassicauda Cancer therapy, Macrophage polarization.

## Abstract

**Objective:**

Colorectal cancer (CRC) is among the deadliest malignancies, often diagnosed at advanced stages, limiting treatment efficacy and necessitating alternative therapeutic approaches. Scorpion venom has emerged as a promising source of bioactive compounds for cancer therapy. This study investigated the anti-cancer potential of *Androctonus crassicauda* scorpion venom fractions against CT-26 colon cancer cells.

**Materials and Methods:**

*A. crassicauda* venom fractions were isolated using gel filtration chromatography. Murine peritoneal macrophages, harvested from BALB/c mice, were polarized towards the M2 phenotype and characterized by flow cytometry. Real-time PCR and ELISA quantified M1 and M2 macrophage-associated gene and cytokine expression. The impact of venom fractions on CT-26 cell proliferation and migration was assessed via MTT and wound-healing assays. Phagocytic activity was evaluated using a yeast phagocytosis assay.

**Results:**

The F2 venom fraction significantly upregulated pro-inflammatory gene and cytokine expression, and downregulated anti-inflammatory gene and cytokine expression in M2 macrophages. Furthermore, the F2 fraction significantly inhibited CT-26 cell proliferation and migration. Critically, it also enhanced the phagocytic capacity of M2 macrophages.

**Conclusion:**

Our results suggest that the F2 fraction of *A. crassicauda* scorpion venom reprograms tumor-associated M2 macrophages towards an anti-tumor M1 phenotype. These findings suggest the potential of the F2 fraction of *A. crassicauda* scorpion venom as a novel therapeutic strategy for the treatment of colon cancer. However, to confirm this potential, further *in vivo* studies need to be carried out.

## Introduction

Cancer is considered a globally prevalent and life-threatening illness that significantly impacts patients, as a result of the notable adverse effects of conventional treatments such as postoperative complications, chemotherapy-related issues, and radiotherapy-induced problems (Anand et al. 2022; Bray et al. 2024). Alternative approaches are crucial to developing effective and safe cancer treatment strategies. Among the effective and alternative methods that have attracted the attention of many researchers in the field of cancer today is the use of anti-cancer peptides obtained from the venom of various animals, including snakes, bees, and scorpions (Badr et al. 2013; Dable-Tupas et al. 2024). Scorpion venom is a complex combination of proteins, peptides, biogenic amines, salts, and mucoproteins (Ryan et al. 2021; Suhas 2022). Increasing experimental evidence supports the anticancer potential of scorpion venom, with crude venom and purified proteins and peptides exhibiting the ability to suppress various hallmarks of cancer in laboratory-based experiments and in living organisms (Bayatzadeh et al. 2020; Jafari et al. 2020). Scorpion venom contains fractions or active peptides (bioactive peptides) that have different functions and structures. These components can serve as effective drugs with potential applications in cancer immunotherapy. Compared to crude venom, other advantages of these refined fractions are reduced side effects, low toxicity, biodegradability, and bioavailability (Ryan et al. 2021; Zerouti et al. 2019). Comprehensive research has demonstrated that venom peptides elicit their pro-inflammatory properties through stimulation of the synthesis of inflammatory cytokines such as interleukin (IL) -1, -6, -8, and -12, tumor necrosis factor-alpha (TNF-α), and interferon-gamma (IFN-γ) (Hadaddezfuli et al. 2015; Reis et al. 2019). Scorpion venom has been shown to influence macrophage inflammatory responses. Accumulating evidence suggests that scorpion venom changes the inflammation of macrophages by making inflammatory substances like IL-1β and TNF-α (Petricevich et al. 2007; Ramírez-Bello et al. 2014; Saidi et al. 2018b). In a study, it was shown that the venom of *Androctonus australis hector* (Aah) scorpion stimulates macrophages to adopt the M1 subset. This occurs through a decreased expression of arginase 1 (Arg-1), an increase in TNF-α levels, and an increased expression of inducible nitric oxide synthase (iNOS) (Ait-Lounis and Laraba-Djebari 2015b). Scorpions of the Buthidae family, including *A. crassicauda*, are dangerous and can pose a significant health risk. These organisms have a broad geographical distribution in the Middle East (Bayatzadeh et al. 2020). The cytotoxic and anticancer properties of the venom of *A. crassicauda* were studied in cultured human neuroblastoma SH-SY5Y and MCF-7 cell lines. After the cells were exposed to scorpion venom, the results indicated that the venom suppressed proliferation through the induction of apoptosis by increasing nitric oxide production, caspase-3 activity, and decreasing mitochondrial membrane potential (Salem et al. 2016; Zargan et al. 2011a). *A. crassicauda* venom has been demonstrated to inhibit cell development by halting the cell cycle in the S phase and causing cell death by depolarizing the mitochondrial membrane (Zargan et al. 2011a). It reduces the viability of mouse “brain” tumor cells (BC3H1) by about 50% after 48 hr of exposure (Caliskan et al. 2013). Furthermore, *A. crassicauda* venom has demonstrated the ability to elicit an enhancement of tumor immunogenicity in CT-26 tumor-bearing mice. This is accomplished by enhancing the synthesis of cytokines, including IL-1 and IFN-γ (Amirgholami et al. 2020). 

The tumor microenvironment (TME) has a complex structure consisting of various cells including immune cells that have crucial functions in the development and progression of tumors (de Visser and Joyce 2023; Pernot et al. 2022). Macrophages are able to infiltrate tumor tissues, therefore, they are one of the most important immune cells in tumor immunogenicity. Macrophages have two primary phenotypes and transitioning between pro-inflammatory M1 and anti-inflammatory M2 phenotypes in response to diverse stimuli (Lendeckel et al. 2022). Researchers have shown that Tumor-associated macrophages (TAMs) have properties associated with the M2 phenotype (Yang et al. 2020). Hence, reprogramming M2 tumor-associated macrophages into M1 anti-tumor macrophages holds promise as a therapeutic strategy to combat tumor growth and proliferation. 

The primary objective of this study was to investigate the anti-tumor capabilities of *A. crassicauda* and the mechanisms involved. Specifically, we assessed the impact of *A. crassicauda* fractions on macrophage polarization. Examination of the anti-tumor effects of reprogrammed macrophages on CT-26 cells was another objective of this study.

## Materials and Methods

### Scorpion venom purification

The* A. crassicauda *venom was acquired from the Razi Institute, Ahvaz branch (Ahvaz, Iran). The venom sample was solubilized in a sterile solution containing 20 millimolar ammonium acetate buffer pH 8.6 ± 0.1(. The solution was centrifuged at 2000 rpm for 10 min to remove mucilaginous and insoluble material. The remaining liquid was collected for further processing. 

### Isolation fraction from venom

**Figure 1:**
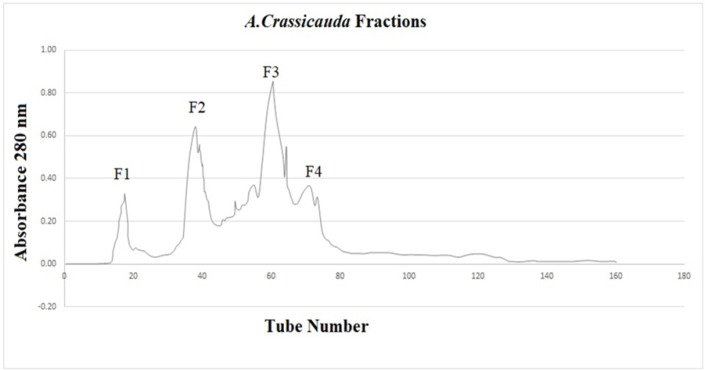
The protein fractions of scorpion venom from A. crassicauda. The gel filtration used a Sephadex G50 column in equilibrium with an ammonium acetate buffer (pH 8.6 ± 0.1) and a flow rate of 60 ml/hr.

Gel filtration chromatography was employed to perform fractionation. The column was washed with a buffer acetate-ammonium (pH 8.6 ± 0.1) at a 60 ml/hr flow rate. The fractions were gathered, and their absorbance was quantified at 280 nm using a spectrophotometer (CamSpec M501, UK). Individual fractions of *A. crassicauda* venom were identified based on their unique absorbance profiles (Figure 1). Protein concentrations in each fraction were then quantified using the Bradford assay (Bradford 1976)

.

### Peritoneal macrophages isolation and induction of M2 macrophages

To recruit macrophages into the peritoneal cavity, 6- to 8-week-old BALB/c mice were administered a single intraperitoneal (IP) injection of 2 ml of 3% thioglycollate broth (Cole-Parmer, Canada). After four days, the mice were euthanized, and the peritoneal cavity lavage fluid was prepared with cold PBS (phosphate-buffered saline) and 3% FBS (fetal bovine serum) (Schneider 2013). The collected peritoneal lavage fluid was centrifuged at 1750 rpm for 10 min at 4°C to pellet the cells. Cells were counted, seeded into cell culture plates containing complete RPMI 1640 medium (supplemented with 15% FBS and 1% penicillin/streptomycin), and incubated for 3 hr at 37°C with 5% CO_2_ and 95% humidity. Following incubation, non-adherent cells were removed during a medium change. The purity of the enriched macrophages was subsequently assessed using flow cytometry analysis with specific antibodies targeting surface markers of CD68 and CD80. To promote the development of the M2 type, the described macrophages were subjected to a 24-hr treatment with IL-4 (60 ng/ml). Subsequently, the expression level of *Fizz-1*,* CD206*, and *Arg-1* (as markers for M2 phenotype), as well as *CD86*, *iNOS*, and *IRF5* (interferon regulatory factor 5) (as markers for M1 phenotype), was determined by qRT-PCR (quantitative real-time polymerase chain reaction).

### Viability assessment of macrophages in vitro

To assess the effects of the *A. crassicauda* fractions on macrophage viability, we used MTT (3- (4, 5-dimethylthiazol-2-yl)-2, 5-diphenyl, tetrazolium bromide) assay. The isolated macrophages were seeded into a cell culture dish at a density of 1 x 10^5^ cells per well. The cells were then exposed to varying concentrations of *A. crassicauda* fractions (5, 10, 25, 50, 100, and 200 μg/ml) at 37°C, 5% CO_2_, and 95% humidity for 24 hr. Following incubation, the media containing the fractions was replaced with a fresh medium. Each well was supplemented with 10 μl of MTT solution (5 mg/ml, pH 4.7 ± 0.1) and subsequently incubated for 4 hr to facilitate MTT reduction. After the supernatant was discarded, each well was treated with 100 μl of dimethyl sulfoxide (DMSO) to dissolve the formazan crystals. The absorbance was measured at 570 nm (ELX800, BioTek, USA). All experiments were done in triplicate. The viability was then calculated in percentage using the following equation.

$[\mathrm{Viability\%}=\frac{\mathrm{ODoftreatedwells}-\mathrm{ODofblankwells}}{\mathrm{ODofcontrolwells}-\mathrm{ODofblankwells}}\times 100$

### Administration of A. crassicauda fractions to M2 macrophages

 To assess the effect of *A. crassicauda* fractions on M2 macrophage function, the cells were placed into a cell culture plate and exposed to varying concentrations of 50 µg/ml F1, 100 µg/ml F2, 50 µg/ml F3, and 100 µg/ml F4 fractions for 24 hr. These concentrations were chosen based on the results of the viability assay. Untreated M2 macrophages were used as the control group.

### Total RNA extraction and qRT-PCR analysis

RNA was isolated using the RNeasy Mini Kit (Qiagen, USA), and the concentration of total RNA in the final eluates was measured using a NanoDrop spectrophotometer device. Extracted RNA was reverse transcribed into cDNA (complementary DNA) using the AddScript cDNA Synthesis kit (Tehran Cavosh Clon, Sina Clon, Tehran, Iran), according to the manufacturer’s instructions. To assess the influence of *A. crassicauda* fractions on the polarization of macrophages, the expression levels of M2 markers (*Fizz-1, CD206, Arg-1*) and M1 markers (*CD86, iNOS, IRF5*) were quantified by qPCR using specific primers and Sina Green HS-Qpcr Mix, 2X kit (Tehran Cavosh Clon), under suboptimal cycling conditions. Furthermore, the results were standardized using beta-2 microglobulin (β2M) as a reference gene and analyzed with the 2^-ΔΔCT^ method (Table 1). 

### Enzyme-linked immunosorbent (ELISA) analysis

Supernatants from cultured macrophages in each treatment group were analyzed for cytokine production using the ELISA method. The levels of IL-10 and TGF-β (transforming growth factor-beta) related to the M2 phenotype as well as IL-1β and TNF-α associated with the M1 phenotype were measured by ELISA kits from the Karmania Parsgene (Iran) according to the manufacturer's instructions. 

### Cell culture

The CT26 colon carcinoma cell line was purchased from the National Cell Bank of Pasteur Institute (Tehran, Iran). The CT26 cells were cultured in RPMI 1640 enriched with 10% FBS (Biosera, France), 1% non-essential amino acids, and 1% Pen-Strep (penicillin/streptomycin) (Biosera, France) at 37°C temperature, 5% CO2, and 95% humidity. The culture medium was replenished every three days to achieve the desired cell density. Upon reaching confluency, the cells were passaged using 0.25% Trypsin-EDTA solution (Bio Idea Co, Iran). This solution gently detached adherent CT-26 cells from the culture flask surface and allowed them to be transferred to fresh culture vessels for continued proliferation.

### MTT assay

CT-26 colon carcinoma cells were cultured in media collected from stimulated and unstimulated macrophages. The assessment of cell viability was conducted via the MTT assay which was performed using an MTT kit (Kiazhen, Tehran, Iran). The conditioned media was applied in triplicate for 24, 48, and 72 hr. Following incubation, the viability of the CT-26 cells was carefully assessed to quantify their response to the conditioned media exposure.

**Table 1:** The primer sequence employed for SYBR Green real-time PCR analysis.

**Gene **	**Forward **	**Reverse **	**Accession number**
*CD86 *	5′-TCAATGGGACTGCATATCTGCC-3′	5′-GCCAAAATACTACCAGCTCACT-3′	NM_019388.3
*iNOS *	5′-GTTCTCAGCCCAACAATACAAGA-3′	5′-GTGGACGGGTCGATGTCAC-3′	NM_001313921.1
*IRF5 *	5′-GGTCAACGGGGAAAAGAAACT-3′	5′-CATCCACCCCTTCAGTGTACT-3′	NM_001252382.1
*Fizz-1 *	5′-AGGAGCTGTCATTAGGGACATC-3′	5′-CCAGTAGCAGTCATCCCAGC-3′	NM_020509.4
*CD206*	5′-CTCTGTTCAGCTATTGGACGC-3′	5′-CGGAATTTCTGGGATTCAGCTTC-3′	NM_008625.2
*Arg-1*	5′-CTCCAAGCCAAAGTCCTTAGAG-3′	5′-AGGAGCTGTCATTAGGGACATC-3′	NM_007482.3
*β2m*	5′-TTCTGGTGCTTGTCTCACTGA-3′	5′-CAGTATGTTCGGCTTCCCATTC-3′	NM_009735.3

### In vitro wound healing assay

A scratch wound healing assay was conducted to assess the effect of macrophage treatment on CT-26 cell migration. A monolayer of CT-26 tumor cells was subjected to a straight scratch inflicted by a pipette tip to create an *in vitro* wound. Following removal of the cellular debris via washing, the wound area was exposed to conditioned media from treated and controlled macrophages in triplicate for 48 hr. Images were obtained using an inverted microscope (Optika, Italy) to observe the movement of the CT-26 tumor cells into the region of injury at three designated points: 0 (immediately after scratching), 24, and 48 hr. ImageJ software (NIH, USA) was then employed to quantify the wound closure percentage. This analysis utilized the following formula:

$\left[\mathrm{Wound closure}\left(\%\right)=\left(1-\left(\frac{\mathrm{Wound Area at T}a_{m}}{\mathrm{Wound area at Ta}}\right)\right)\times \mathrm{100}\right]$

Ta represents the time point directly after scratching, and T$a_{m}$ signifies the time following wounding (where m = 24 and 48 hr).

### Phagocytosis assay

Here, a yeast phagocytosis assay was employed to determine the phagocytic capacity of macrophages from different treatment groups. Briefly, the macrophages were incubated with RPMI 1640 medium containing yeast particles at a 10:1 ratio (macrophages: yeast) for 1 hr. The yeast challenged the macrophages, incubated for 30 min at 37°C and 5% CO₂. The cells were then washed with PBS to eliminate any unbound yeast, fixed with methanol, stained with Wright-Giemsa stain, and visualized using an inverted microscopy (Zeiss Jena, Germany). A total of 500 cells were counted to evaluate the percentage of phagocytic cells. 

### Statistical analysis

GraphPad Prism 8.4.3 software (GraphPad, CA, USA) was used for statistical analysis. The data is presented as the mean ± standard deviation (SD) to provide a clear and understandable presentation. To determine significantly different relationships between groups, we utilized one-way ANOVA, two-way ANOVA, and unpaired t-tests. Statistical significance was defined as p < 0.05. This approach provided robust statistical evaluation of the experimental results.

## Results

### Separation of fractions from A. crassicauda scorpion venom

Gel-filtration chromatography was employed using a Sephadex G50 column to isolate fractions from the venom sample. Investigating the peaks of absorbance at 280 nm revealed the presence of four distinct fractions, designated as F1, F2, F3, and F4 (Figure 1). The protein content of each fraction was subsequently quantified utilizing the BCA assay kit (Parstous Biotechnology, Iran). Fractions F1, F2, F3, and F4 exhibited protein concentrations of 7.5, 6.0, 7.0, and 6.5 mg/ml, respectively.

### Flow cytometry for characterization of the potential of macrophages

**Figure 2:**
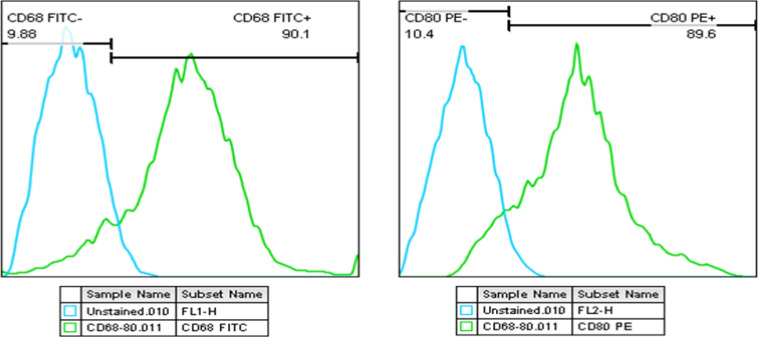
The analysis of flow cytometry. The result shows a significantly high expression of CD68 and CD80.

Flow cytometry was employed to characterize isolated peritoneal macrophages by analyzing the surface expression of established macrophage markers CD68 and CD80. As shown in Figure 2, a substantial percentage of cells exhibited co-expression of CD68 (90.1%) and CD80 (89.6%), indicating the presence of a macrophage population in the isolated sample.

### MTT viability assay for assessment of cytotoxicity of A. crassicauda fractions

**Figure 3:**
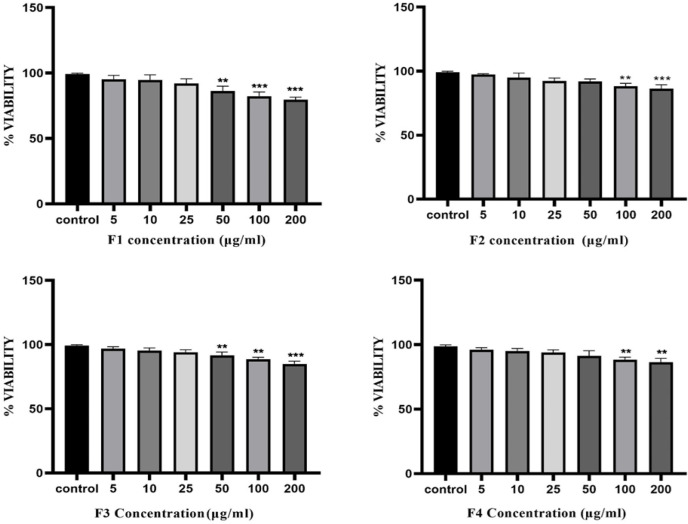
Effect of A. crassicauda fractions on macrophage viability. Macrophages were incubated for 24 hr with various concentrations (5, 10, 25, 50, 100, and 200 µg/ml) of each fraction. The statistical analysis showed mean±SD **p<0.01, *** 0.001 significant differences compared to the control group. (n=3).

MTT assay used to assess the cytotoxic effects of *A. crassicauda* fractions on isolated macrophages. Fractions F2 and F4 exhibited a higher impact on macrophage viability concentrations of 25- 200 µg/ml (Figure 3), these fractions caused a significant decrease in cell viability compared to the control group. Fractions F1 and F3 displayed a similar trend, with no cytotoxic effects observed at concentrations below 25 µg/ml. Based on these findings, subsequent polarization studies employed F1, F2, F3, and F4 at 50, 25, 50, and 25 µg/ml concentrations, respectively. This approach ensured that the chosen concentrations would effectively modulate macrophage activity without inducing substantial cell death.

### Effective polarization of M2 macrophages by IL-4

**Figure 4:**
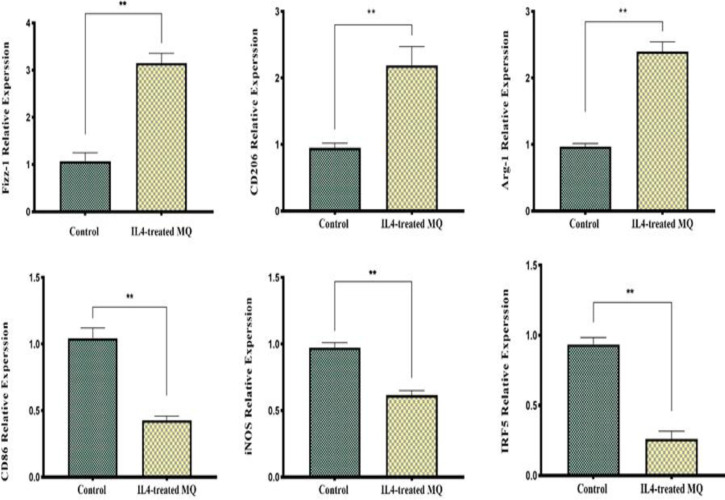
M2 cell polarization after IL-4 therapy. After 24 hr of exposure to IL-4 (60 ng/ml), peritoneal macrophages were examined for the relative expression of M2 markers Fizz-1, CD206, and Arg-1 and M1 markers CD86, INOS, and IRF5. Fold changes were calculated and are presented as mean±SD (n = 3). (**p<0.01, ***p<0.001). (MQ = macrophage).

To promote M2 polarization, macrophages were stimulated with IL-4 for a duration of 24 hr before gene expression analysis. As depicted in Figure 4, this treatment led to a significant upregulation of M2-associated genes, such as *Fizz-1*, *Arg-1*, and *CD206*, relative to untreated controls. In contrast, the expression of factors associated with M1 macrophages, such as *CD86*, *iNOS*, and *IRF5,* was significantly reduced in macrophages activated with IL-4. These data show that macrophages were successfully polarized towards the M2 phenotype in response to IL-4 activation.

### F2 fraction of A. crassicauda scorpion venom induces M1 polarization in macrophages

**Figure 5:**
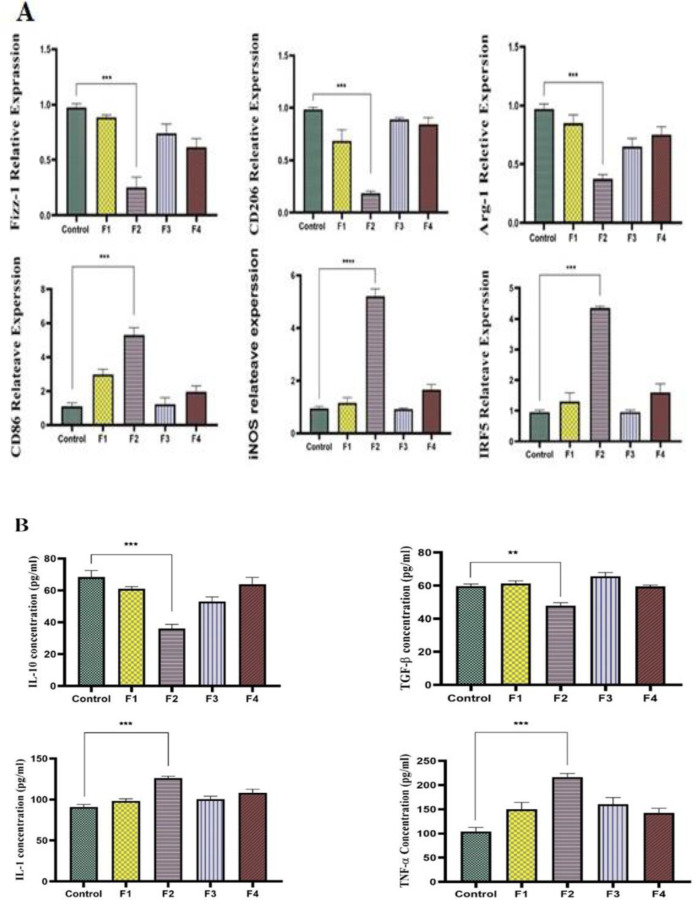
The expression of genes and cytokine levels of M2- and M1-specific markers in macrophages treated with all fractions of A. crassicauda. A) The F2 fraction treatment resulted in a remarkable drop in M2 markers (Fizz-1, CD206, and Arg-1) and a considerable increase in M1 markers (CD86, INOS, and IRF5). B) The secretion of IL-10 and TGF-β (M2 indicators) was drastically decreased, while the production of IL-1β and TNF-α (M1 markers) was greatly enhanced in macrophages treated with F2. Gene expression: the data is adjusted to β2m, and all data is shown as the mean ± SD (n = 3). (**p<0.01, ***p<0.001, ****p<0.0001). (M1 = macrophage type 1), (M2 = macrophage type 2).

The influence of *A. crassicauda* fractions on the repolarization of macrophages from M2 toward M1 was examined. Macrophages that were polarized by IL-4 were subjected to all isolated fractions, and the expression of M1 and M2 markers was assessed using real-time PCR. As shown in Figure 5A, treatment with the F2 fraction resulted in a significant upregulation of M1 markers *CD86* (p 0.001),* iNOS* (p <0.0001), *IRF5* (p <0.001) and a concomitant downregulation of M2 markers *Fizz1* (p<0.001), *CD206 *(p <0.001), and *Arg-1* (p<0.001). In contrast, fractions F1, F3, and F4 did not elicit substantial changes in marker expression. Cytokine secretion was measured using ELISA to further substantiate the M1-promoting effect of F2. In addition, F2 treatment led to a substantial decrease in the M2-related cytokines IL-10 (p <0.001) and TGF-β (p <0.01), whilst M1-related cytokines IL-1β (p <0.001) and TNF-α (p <0.001) exhibited a significant upsurge compared to the control and other treatment groups (Figure 5B). Notably, the remaining fractions F1, F3, and F4 did not cause significant cytokine level changes compared to the untreated group.

### Anti-tumor effects of F2 fraction-treated macrophage-conditioned media on CT-26 cell

**Figure 6:**
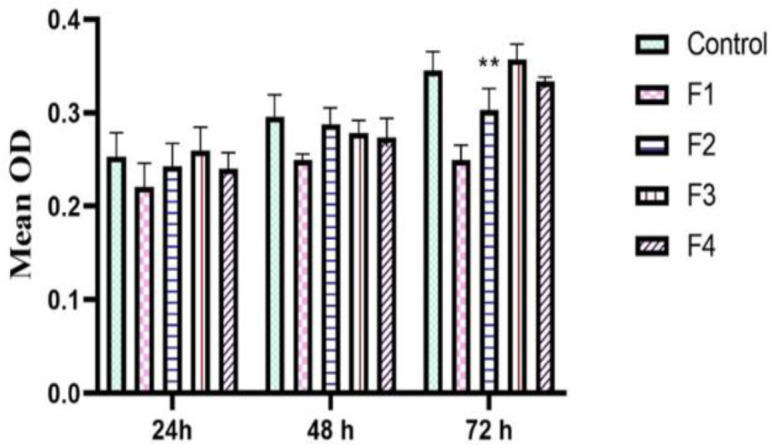
MTT results after CT-26 exposure to conditioned media from F1-, F2-, F3-, and F4-treated macrophages versus control untreated CT-26 cells. F2-treated macrophages had lower proliferative activity in their conditioned media compared to the untreated group (n = 3) (**p≤0.01 as compared to the control group).

The impact of conditioned media from treated macrophages on CT-26 cell proliferation was evaluated using the MTT assay. As shown in Figure 6, F2-treated macrophage supernatant significantly reduced CT-26 cell growth 72 hr post-exposure compared to the control group and groups treated with other venom fractions (p 0.01). This finding indicates that the F2 fraction may have anti-proliferative capabilities against CT-26 colon carcinoma cells. Interestingly, conditioned media from macrophages treated with F1, F3, and F4 fractions did not significantly affect CT-26 carcinoma cell proliferation compared to untreated cells.

### CT-26 cell migration evaluation

**Figure 7:**
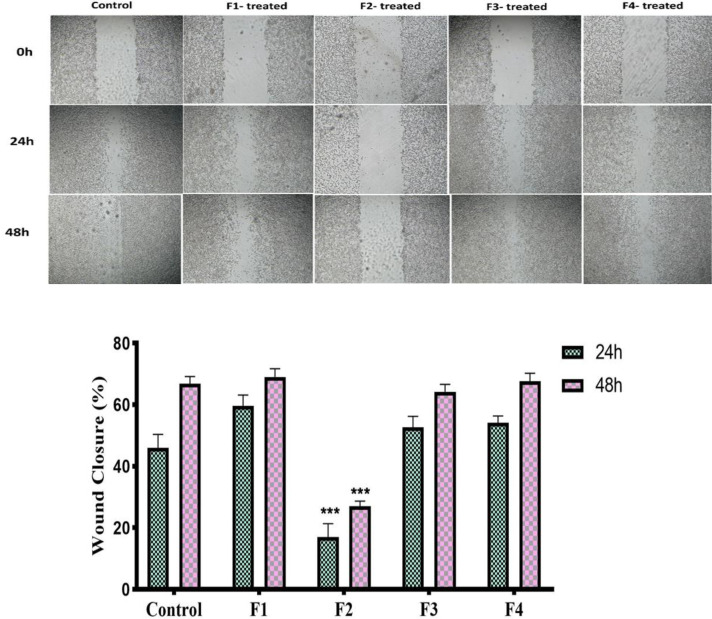
Wound healing analysis. A scratch experiment was performed on CT-26 cells using conditioned media with macrophages treated with different A. crassicauda fractions. CT-26 cells exposed to F2-treated macrophages had lower migration and wound closure rates (n =3 ***p<0.001 as compared to the control group).

To evaluate the anticancer potential of *A. crassicauda* venom fractions, we assessed their ability to inhibit CT-26 carcinoma cell migration using a wound healing assay. All tumor processes, including proliferation and invasion, are associated with increased tumor cell migration. Our results showed that the wound healing process was significantly impeded by the conditioned media derived from macrophages treated with F2, both at 24 and 48 hr, compared to the control and other treatment groups (p <0.0001) (Figure 7). However, the media obtained from macrophages treated with F1, F3, and F4 did not significantly impact the movement process of CT-26 carcinoma cells.

### Macrophage phagocytic activity

**Figure 8:**
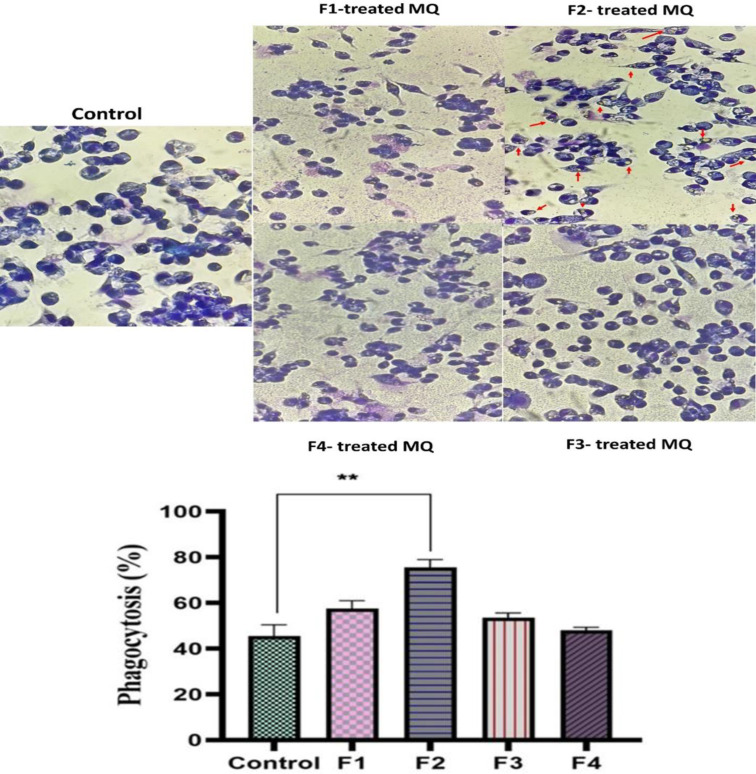
Yeast phagocytosis analysis. The result show an increase in yeast phagocytosis in F2 fraction-treated macrophages compared to the control and experimental groups (n = 3). The flashes in the Figure show phagocyted yeasts.

The phagocytic activity of macrophages following treatment was evaluated by exposing them to yeast particles at a 1:10 ratio. The F2 fraction significantly enhanced macrophages' ability through phagocytosis (Figure 8). F2-treated macrophages exhibited a phagocytosis rate of 78%, (p0.01), which was considerably higher compared to the untreated and other treatment groups. Interestingly, macrophages treated with F1, F3, and F4 fractions displayed a phagocytic activity similar to that of control cells.

## Discussion

TME is a very complex and dynamic environment that is made up of various cells including tumor cells, stromal cells, and immune cells which play a vital role in the development of cancer (de Visser and Joyce 2023). Macrophages are one of the main cells that infiltrate into the tumor environment. M1 macrophages participate in the antitumor response by secreting cytokines, such as IL-1 and TNF- (Genin et al. 2015). In the tumor site, release of cytokines, including IL-13 and IL-4, induces polarization toward M2-type macrophages (Seif et al. 2019). M2 macrophages secrete anti-inflammatory and immunosuppressive cytokines and promote tumor progression (Mantovani et al. 2002). Tumor-associated macrophages (TAM) typically have an M2 phenotype. As a result, repolarization of M2 to M1 is a promising target in cancer treatment. Results of the current study exhibited a substantial influence of the F2 fraction extracted from the venom of *A. crassicauda* scorpion on the polarization of macrophages. A pro-inflammatory M1 phenotype was specifically induced in macrophages by the F2 fraction. Furthermore, we investigated the capacity of the reprogrammed macrophages to suppress the growth and movement of the CT-26 carcinoma cell line, thereby assessing their anti-tumor potential. 

Numerous studies on the venom of different scorpions have demonstrated the ability of venom-derived proteins and peptides to restrict metastasis, induce apoptosis in malignant cells, and decrease their growth. (Aissaoui-Zid et al. 2021; Das et al. 2021; RAVE et al.). The anti-cancer properties of scorpion venom have been observed in several types of tumors, such as breast cancer, gliomas (RAVE et al.), colorectal cancer (Hua et al. 2011), prostate cancer, and hepatocellular carcinoma (Anand et al. 2019a; Anand et al. 2019b). 

Scorpion venom, once feared as a deadly weapon, is now gaining recognition as a source of promising molecules for drug discovery. This venom is a complex mixture of various molecules including lipids, peptides, enzymes, and amino acids, each with the potential to exhibit unique biological functions (Ding et al. 2014; Ortiz et al. 2015). These molecules' structural and functional diversity makes them attractive templates for designing and developing new drugs. One intriguing aspect of scorpion venom research is its impact on macrophage function. Macrophages are crucial immune cells that are pivotal in innate and adaptive immunity and have critical roles in tumor biology. Studies have shown that scorpion venom can direct macrophage polarization towards the M1 subset which is associated with inflammatory and anti-tumor functions. *Androctonus australis hector* (Aah) scorpion venom strongly causes macrophages to shift into the M1 subgroup. As per the findings of Ait-Lounis and Laraba-djebari (2015), when *Androctonus australis hector* venom (*AahV*) is administered as a stimulus, it causes a decrease in the expression of M2-associated genes (IL-10 and *Arg1*) while increasing the expression of mediators of inflammation genes such as *Nos2*, *1L-23*, and *IL-1,* which promote M1 (Ait-Lounis and Laraba-Djebari 2015a). For instance, Saidi et al. (2018) reported that *AahV* scorpion venom induces lung inflammation including alveolar macrophage activation and release of inflammatory mediators (Saidi et al. 2018a). Similarly, the isolation of venom fractions from various *Tityus* species has been shown to augment inflammatory mediator production by macrophages through interactions with CD14, TLR2 (toll-like receptor), and TLR4 receptors (Baradaran and Pashmforoosh 2023; Casella-Martins et al. 2015; Zoccal et al. 2014). 

Our study was performed on different fractions of *A. crassicauda* venom and the results indicate that the F2 fraction of the scorpion venom notably enhances the secretion of IL-1β and TNF-α by peritoneal macrophages. These observations align with previous studies documenting the pro-inflammatory effects of scorpion venom on immune cells (Adi-Bessalem et al. 2015; Fukuhara et al. 2003; Petricevich 2010). Interestingly, recent research has also unveiled the potential anti-inflammatory properties of scorpion venom components. For example, BmKK2, a substance that blocks the Kv1.3 channel, is derived from the venom of the *Buthus martensii Karsch* (BmK) scorpion. BmKK2 has decreased the inflammatory responses of macrophages (Wang et al. 2023). BmKK2 exerts its anti-inflammatory effects by suppressing the *NF-κB*-NLRP3 signaling pathway, which reduces the synthesis of inflammatory mediators. These findings highlight the multifaceted nature of scorpion venom's effects on immune cell function and underscore the need to explore its potential therapeutic applications further.

Similarly, another study showed that scorpion venom polypeptide (SVP) inhibited M2 polarization in alveolar macrophages by blocking the JAK/STAT6 pathway, reducing fibrosis and damage. SVP also reduced M2-related cytokine (IL-4) and TGF-β (Ling et al. 2019; Xu et al. 2022). According to this study, SVP showed a regulatory effect on various immune cells, particularly influencing macrophage polarization towards the M2 phenotype. These findings are consistent with our findings. Sadeghi et al. in a study on the f1 fraction isolated from the venom of *Mesobuthus eupeus*, showed that this fraction inhibited colon cancer cells by increasing M1 markers (Sadeghi et al. 2024). The results of other studies showed differences in the physiological properties of specific fractions of each scorpion venom. The F2 fraction of *A. crassicauda* venom significantly increased the M1 phenotype markers (*iNOS*, *CD86*, and *IRF5*) and decreased the M2 phenotype markers (*Fizz-1*, *Arg-1*, and *CD206*) in macrophages. This indicates a successful transition from the M2 phenotype to the M1 phenotype. The whole *A. crassicauda* venom enhances caspase-3 activity, induces S-phase cell cycle arrest, and induces S-phase membrane depolarization (Zargan et al. 2011a; Zargan et al. 2011b). It also triggers mitochondrial membrane depolarization, reduces cellular movement, and inhibits colony formation.* A. crassicauda* venom has shown an inhibitory effect on various types of cancer observed in the human population (Al-Asmari et al. 2018; Rapôso 2017; Salem et al. 2016; Zargan et al. 2011a; Zargan et al. 2011b). *In vivo* studies further support its anti-tumor effects. In a mouse model, treatment of CT-26 cancer cells with *A. crassicauda* venom enhanced the production of anti-tumor mediators (IL-12, IL-1β, and IFN-γ) and significantly suppressed the proliferation of colon tumor cells in a previous study (Amirgholami et al. 2020). However, this study employed crude venom without fractionation and did not investigate the venom's effect on the polarization of macrophages toward the anti-tumor phenotype. Macrophages are the predominant cell type in the TME and play a critical role in eliminating cancer cells, potentially serving as a therapeutic immunomodulator. Our research aligns with this study and extends its findings. Also, similar to our study, the mentioned study evaluated CD markers of T cells and inflammatory cytokines.

First, we established the ability of the F2 fraction extract from *A. crassiauda* venom to induce M2 macrophages to transform into M1 macrophages, then we performed *in vitro *studies to assess M1 macrophage migration and tumor cell proliferation. Consistent with our previous findings, we observed a significant reduction in CT-26 tumor cell migration and proliferation induced by macrophages polarized with F2.

Findings from our study demonstrated scorpion venom's anticancer effects on colorectal cancer. The current research supports the effectiveness of the F2 fraction from *A. crassicauda* venom in macrophage repolarization to the shift from M2 macrophages (pro-tumoral) to M1 macrophages (anti-tumoral). Furthermore, our *in vitro* study demonstrated that macrophage repolarization facilitated by the F2 fraction of *A. crassicauda* venom may significantly suppress tumor proliferation and migration. As a result, using scorpion venom to induce M2 to M1 macrophage polarization has the potential to be an innovative therapeutic approach for the development of anticancer biotherapeutic drugs. However, more detailed and complete research is needed to fully understand the mechanisms involved in this special property of scorpion venom. Furthermore, it would be interesting to explore the impact of scorpion venom on other immune cells and non-immune ones in the TME. This could be achieved by conducting comprehensive *in vivo* research on animal models.

## Data Availability

Data will be made available on request.
